# Diseases Peculiar to Bakers

**Published:** 1886-10

**Authors:** 


### Diseases Peculiar to Bakers.

(Translated from the German.)

Investigations are now being made in regard to diseases peculiar to those engaged in work as bakers; the first impulse to the investigation was given at the Dental Institute at Leipsic. One of the faculty says: "I have the opportunity to examine a great many patients from among mechanics and laborers and nothing surprises me so much as the bad condition of the dental organs of our bakers. The teeth are attacked by dental caries to such a degree that since I have noted this fact, I am able in nearly every case to judge by the condition of the teeth the trade the patient follows. This caries is soft and rapid in progress, its favorite localities are the labial and buccal surfaces. Its tendency is towards great penetration and spreads over the whole surface attacked. Commences at the gingival margin and works its way rapidly toward the cutting edge, rather than upon the root. All the patients I examined were aged between 17 and 23 years, a period at which the teeth should be at their best. There seems to be no doubt but that we have a disease peculiar to bakers. During their work more or less flour is inhaled and settles upon the exposed surfaces of the teeth, and it is not to be supposed that people whose day's work begins at 2 a.m. give their teeth proper attention; it is questionable whether it would prevent the decay, as during their hours of labor they are exposed to the destructive action of the flour dust. I have also noticed the same state of affairs, though to a lesser degree, in the mouths of the children of confectioners. It is very likely that millers will be found afflicted to a greater degree than the bakers, that point being now under investigation.

